# Biofilm-Forming Abilities of *Listeria monocytogenes* Serotypes Isolated from Different Sources

**DOI:** 10.1371/journal.pone.0137046

**Published:** 2015-09-11

**Authors:** Swapnil P. Doijad, Sukhadeo B. Barbuddhe, Sandeep Garg, Krupali V. Poharkar, Dewanand R. Kalorey, Nitin V. Kurkure, Deepak B. Rawool, Trinad Chakraborty

**Affiliations:** 1 ICAR Research Complex for Goa, Old Goa 403 402, India; 2 Department of Microbiology, Goa University, Taleigaon Plateau, Goa 403 206, India; 3 Department of Microbiology and Animal Biotechnology, Nagpur Veterinary College, Maharashtra Animal and Fishery Sciences University, Nagpur 440006, India; 4 Division of Veterinary Public Health, Indian Veterinary Research Institute, Izatnagar, 243122, India; 5 Institute of Medical Microbiology, Justus-Liebig University, 35392 Giessen, Germany; 6 National Institute of Biotic Stress Management, IGKV Campus, Krishak Nagar, Raipur, Chhattisgarh, 492012, India; University of Hyderabad, INDIA

## Abstract

A total of 98 previously characterized and serotyped *L*. *monocytogenes* strains, comprising 32 of 1/2a; 20 of 1/2b and 46 of 4b serotype, from clinical and food sources were studied for their capability to form a biofilm. The microtiter plate assay revealed 62 (63.26%) strains as weak, 27 (27.55%) strains as moderate, and 9 (9.18%) strains as strong biofilm formers. Among the strong biofilm formers, 6 strains were of serotype 1/2a and 3 strains were of serotype 1/2b. None of the strain from 4b serotype exhibited strong biofilm formation. No firm correlation (p = 0.015) was noticed between any serotype and respective biofilm formation ability. Electron microscopic studies showed that strong biofilm forming isolates could synthesize a biofilm within 24 h on surfaces important in food industries such as stainless steel, ceramic tiles, high-density polyethylene plastics, polyvinyl chloride pipes, and glass. Cell enumeration of strong, moderate, and weak biofilm was performed to determine if the number of cells correlated with the biofilm-forming capabilities of the isolates. Strong, moderate, and weak biofilm showed 570±127× 10^3^ cells/cm^2^, 33±26× 10^3^ cells/cm^2^, 5±3× 10^3^ cells/cm^2^, respectively, indicating that the number of cells was directly proportional to the strength of the biofilm. The hydrophobicity index (HI) analysis revealed higher hydrophobicity with an increased biofilm formation. Fatty acid methyl esterase analysis revealed the amount of certain fatty acids such as iso-C15:0, anteiso-C15:0, and anteiso-C17:0 fatty acids correlated with the biofilm-forming capability of *L*. *monocytogenes*. This study showed that different strains of *L*. *monocytogenes* form biofilm of different intensities which did not completely correlate with their serotype; however, it correlated with the number of cells, hydrophobicity, and amount of certain fatty acids.

## Introduction

*Listeria monocytogenes* is a gram-positive bacterium, emerging as a foodborne pathogen and the etiological agent of listeriosis. Listeriosis most commonly found in immuno-compromised individuals, neonates, pregnant women, elderly persons, and AIDS patient [1, 2]. The infection is characterized by various clinical conditions such as spontaneous abortions, meningoencephalitis, septicaemia, gastroenteritis, and serious infections to the newborns [3, 4]. Although less common, *L*. *monocytogenes* infection is a serious problem because it exhibits a high case-fatality rate (30%), hospitalization rate (91%), and neonatal death rate (50%) [5]. Since the last two decades, there is a considerable increase in the incidences of listeriosis [6–8].

The consumption of contaminated food is the maincause of *L*. *monocytogenes* infection. Among the foods, the ready-to-eat (RTE) foods, which are industrially processed and require storage at low temperature, frequently get linked to listeric infections [9]. The major reason for the contamination of these industrially processed foods appears to be the persistence of *L*. *monocytogenes* at food processing environment [10]. The organism may enter in the food processing environment through several routes, and it may get established [11]. In addition, for *L*. *monocytogenes*, such activities supported by factors such as growth at the wide pH range, salt tolerance, growth at low temperature, resistance to different stress conditions and biofilm formation ability. *L*. *monocytogenes* has been shown to occur on the surfaces in food industries that may directly or indirectly come in contact with the food leading to the contamination. Several such industrially processed foods, such as cheese, meat, have been reported to be contaminated with *L*. *monocytogenes* [12, 13]. *L*. *monocytogenes* biofilms have been observed to resist cleaning, disinfectant, desiccation, and UV light, enhancing its probability to persist [14, 15]. Subtyping of *L*. *monocytogenes* has played an important role in the epidemiology of *L*. *monocytogenes* [16, 17]. Several researchers have tried to relate *L*. *monocytogenes* serotypes with their abilities to adhere, to form biofilm, to resist disinfectant or antibiotic, and to tolerate stress [18]; however, certain results remain contradictory or inconclusive [19–21]. In contrast, Weiler et al. [22] suggested that biofilm formation and attachment of *L*. *monocytogenes* was strain specific rather than serotype specific. Overall, the data varied, apparently depending upon the isolates, and therefore, no clear correlation with serotypes or lineages could be established to date [23, 24]. Therefore, the analysis of *L*. *monocytogenes* isolates from diverse sources having different genetic make ups is necessary to deduce a relation, if any, between biofilm formation and serotypes.

Unlike other strong biofilm formers such as *Pseudomonas* spp. or *Staphylococcus* spp., *L*. *monocytogenes* did not produce sufficient extra polymeric substances although it is known to form three-dimensional films [23]. Therefore, it is obvious that *L*. *monocytogenes* must possess some other abilities to form biofilms. To date, there is no strong evidence showing the exact mechanism of biofilm formation by *L*. *monocytogenes*. Recent studies showed that the amount and type of fatty acids played a differential role influencing the biofilm-forming properties of bacteria [25–27]. Fatty acids in *L*. *monocytogenes* have been studied with respect to adaptation to cold temperature [28]. However, adequate studies have not been performed profiling fatty acids among different biofilm formers. In addition, microbial adherence is largely dependent upon the surface charge and hydrophobicity [29–31]. Because *L*. *monocytogenes* has limited biofilm-forming accessories, the role of cell surface hydrophobicity for adherence and biofilm formation requires particular attention.

At ‘Indian *Listeria* Culture Collection’ (ILCC), we have a collection of *L*. *monocytogenes* strains belonging to different serotypes which are obtained from various sources such as food and food industries, environmental, human, and animal clinical cases. In this study, we assessed the biofilm-forming ability of *L*. *monocytogenes* strains of different serotypes.

## Materials and Methods

### Bacterial strains and serotypes

A total of 98 previously characterized and serotyped *L*. *monocytogenes* strains isolated from various sources were included in this study (S1 Table). All the isolates were obtained from Indian Listeria Culture Collection (ILCC) center, Goa. Of these 98 isolates, 32 were of 1/2a serotype, 20 were 1/2b serotype, and 46 were 4b serotype. These strains were isolated from clinical and food sources (milk and milk products: 34, meat and meat products: 14, human clinical cases: 18, and animal clinical cases: 32). Isolates maintained in brain heart infusion (BHI) broth with 15% glycerol stock were revived on PALCAM agar. The strains were subjected to biofilm formation, and related assays were performed.

### Microtiter plate assay

Microtiter plate assay was performed as previously described by Borucki [21]. Overnight grown listerial culture (200 μl) was transferred into 7 wells of a column of sterile polyvinyl chloride (PVC) microtiter plate (GenAxy). The eighth well of the column was kept as a control. Plates were covered with sterile lid and edges of the plate were then sealed with parafilm. The plates were incubated for 24 h at 37°C with 150 rpm shaking. After 24 h, the cell turbidity was measured using a microtiter plate reader (Multiscan Ascent, Thermofisher), at an optical density (OD) at 595 nm (OD_595_). The liquid from each of the wells was removed, and unattached cells were removed by rinsing three times in 250 μl of sterile water. Plates were then dried in an inverted position for 30 min. Biofilms were stained by adding 200 μl of 0.1% crystal violet (CV) solution (in sterile water) to each well; plates were sealed by parafilm and incubated for 45 min at room temperature. Unbound dye was removed by rinsing three times in 250 μl of sterile water. The CV was solubilized by adding 210 μl of 95% ethanol and incubated at 4°C for 30 min. The contents of each well (200 μl) were then transferred to a sterile polystyrene microtiter plate, and OD_595_ of each well was measured by microplate reader. Final OD for the turbidity and crystal violet was calculated by subtracting the OD of the control wells from the average OD of seven test wells. Statistical analysis was performed using one-way ANOVA test.

### Quantitative biofilm formation assay

The quantitative biofilm formation assay was performed to enumerate the listerial cells as described by Jeyasekaran et al. [32]. Representative six isolates from each group—strong (ILCC-297, 306, 312, 395, 540, 400), medium (ILCC- 041,099, 177, 289, 302, 557), and weak (ILCC- 496, 567, 559, 289, 163, 535) biofilm formers were selected for study. Clean, grease-free glass slides were placed in 100 ml screw-cap bottles containing 48 ml of BHI broth and autoclaved. The medium was inoculated with 2 ml of cultures overnight grown in BHI broth and incubated for 24 h at 37°C under shaking condition at 150 rpm. The glass slides were aseptically removed and washed in sterile phosphate buffered saline (PBS) to remove unattached cells. The cells were removed by rubbing with a sterile cotton swab (Hi-Media). The swab was then transferred to 10 ml PBS containing 0.01% of Tween20, shaken vigorously, and serial ten fold dilutions of each strain were plated on BHI agar. To limit variation in the data because of incomplete removal of the cells from the glass slides, multiple swabs were used for the same area and inoculated in PBS containing 0.01% of Tween 20. The experiment was repeated three times. Colony count was performed and calculated for cells in biofilm/cm^2^.

### Scanning Electron Microscopy

Scanning electron microscopy (SEM) was performed to observe the biofilm formation at different time intervals and on different industrially important surfaces. The biofilm formation of *L*. *monocytogenes* at different time intervals was studied on the glass slide. Four sets were prepared with clean, grease-free glass slide in 100 ml glass bottle containing 40 ml of BHI broth; bottles were plugged and autoclaved. An overnight grown strong biofilm former strain was inoculated (2 ml), and respective bottle sets were incubated at 37°C for 2, 6, 12, and 24 h. Slides were removed and washed three times with PBS to remove the unattached cells. Slides were subsequently dried and used for SEM.

Stainless steel (SS304), PVC, and tiles used in the industry were obtained and cut in 2 × 4 mm size, whereas, for glass, glass slides were used. This material was obtained from the food-processing industrial equipment manufacturer. Coupons were inserted in the screw-cap bottle, and the assembly was autoclaved. The best biofilm-forming listerial strain observed in the study (ILCC306) was further considered for SEM study. Overnight grown culture (2 ml, in BHI broth) was poured in glass bottles containing 48 ml of BHI broth and incubated at 37°C for 24 h under shaking (150 rpm) conditions. Coupons were removed and gently washed with 5 ml of PBS three times and dried for SEM analysis. The samples were fixed by using 2% glutaraldehyde in sodium-cacodylate buffer (0.1 M, pH 7.2). Post-fixation was performed in 1% osmium tetroxide for 1 h at 25°C and dehydrated in a graded series of ethanol (40%, 60%, 80%, 95% and 100%). Dried coupons were sputter—coated with gold and visualized under SEM (JEOL, Model: 5800LV, Japan).

### Determination of hydrophobicity

The hydrophobicity index (HI) of strong, moderate, and weak biofilm-forming strains was determined by microbial adherence to n-hexadecane (MATH) test as described by Di-Bonaventura [33]. In brief, an overnight (18 h) culture (4 ml) was centrifuged at 8,000 × *g* for 5 min. Cell pellets were washed three times using PBS and re-suspended in the same (4 ml). The growth OD was adjusted using PBS at a constant value (A_0_). Later, 1 ml of n-hexadecane (Sigma—Aldrich) was added and vortexed for 1 min. The suspension was allowed to separate for 15 min at room temperature. Approximately 200 μl of cell suspension was transferred to each well of a microtiter plate and turbidity of the cell suspension was measured at 600 nm using a microtiter plate reader. The HI was calculated as follows: 100 × (1−A_1_/A_0_), where A_0_ and A_1_ are the initial and final ODs of the aqueous phase, respectively. The experiment was repeated thrice for consistency.

### Analysis of *L*. *monocytogenes* fatty acid profiles

Each of the 6 isolates of strong, moderate, and weak biofilm-forming capability were analyzed to determine the variation in fatty acid profile as described by Whittaker et al.[34], using fatty acid methyl ester (FAME) analysis. In brief, using a sterile disposable wooden stick, 40–50 mg of bacterial growth from tryptose soy agar was harvested in a sterile screw-cap tube. The cellular fatty acids were saponified by adding 1ml of 3.75 N NaOH in aqueous methanol and heated in a boiling water bath for 30 min. For methylation of the fatty acids, 2.0 ml of 3.25 N HCl in methanol was added, and the tubes were heated at 80°C for 10 min. FAMEs were extracted with 1.25 ml of 1:1 hexane/methyl tert-butyl ether. The organic phase was washed with 3.0 ml of 0.3 N NaOH, separated, and measured by the gas chromatography system (Agilent 6850). Comparing the isolates with reference standards, total fatty acids ranging from C_12:0_ to C_20:0_ were identified and their relative amounts were estimated by Sherlock microbial identification system.

## Results

### Microtiter Plate assay

**Fig 1:**
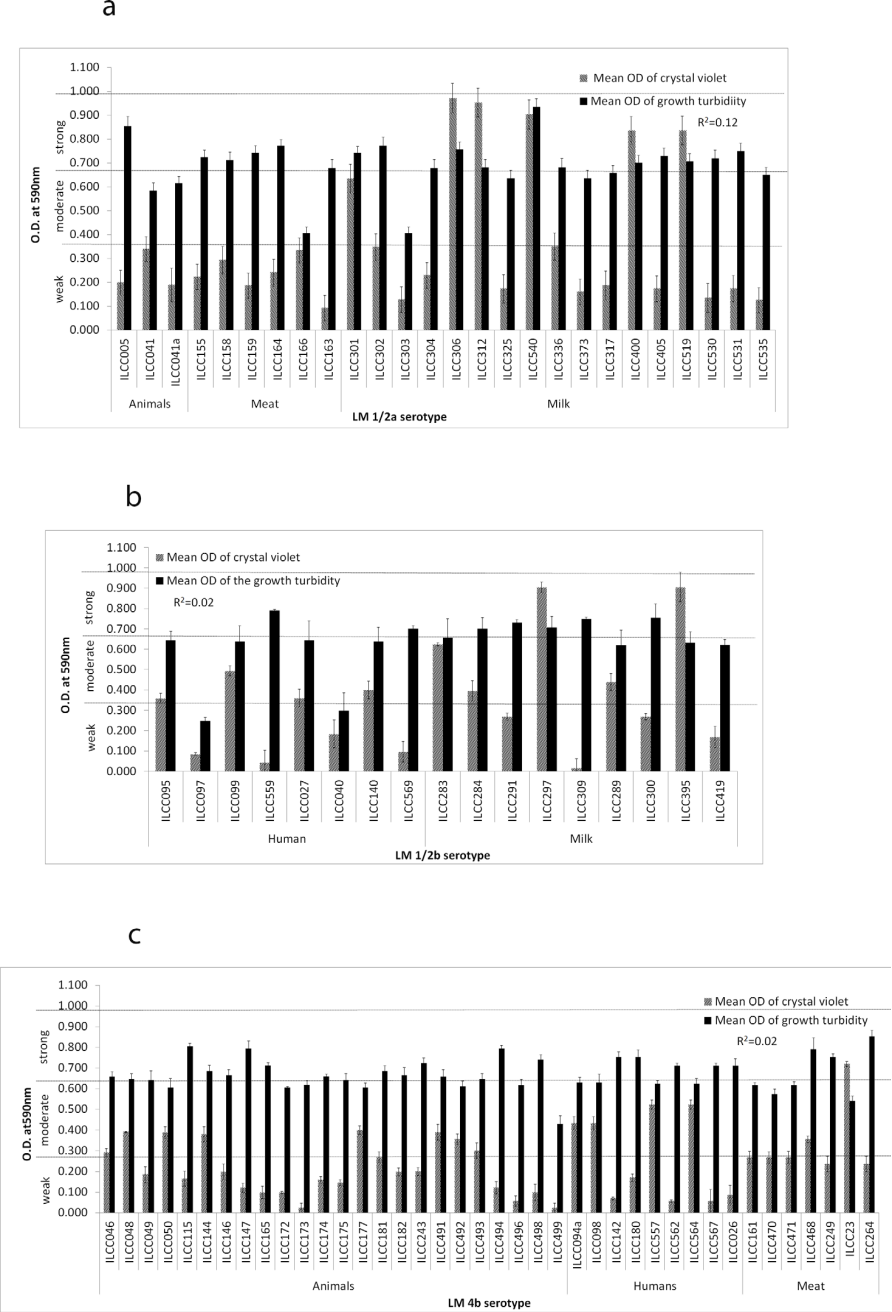
Biofilm formation and growth ability of *L*. *monocytogenes* strains of different serotypes. **(a)** OD_595_ of the biofilm after staining with crystal violet, and growth turbidity of the strains belonging to serotype 1/2a; **(b)** OD_595_ of the biofilm after staining with crystal violet, and growth turbidity of the strains belonging to serotype 1/2b; **(c)**. OD_595_ of the biofilm after staining with crystal violet, and growth turbidity of the strains belonging to serotype 4b.

A total of 98 listerial strains were analyzed for their biofilm-forming ability using the microtiter plate assay. Strains were compared as previously described by Harvey et al. [35] and arbitrarily designated as weak (OD_595_<0.323), moderate (OD_595_ = 0.324–0.648), or strong biofilm formers (OD_595_>0.648). Of 98, 62(63.26%) were assessed as weak or non-biofilm formers, 27 (27.55%) were moderate biofilm formers and 9 (9.18%) were strong biofilm formers. Average turbidities of growth and crystal violet stain (the indirect assessment of biofilm-forming ability) of *L*. *monocytogenes* estimated after 24 h incubation at 37°C are shown in Fig 1a, 1b and 1c.

No serotype was found to be predominant for the biofilm formation (P > 0.05). Of 32 serotype 1/2a strains, 6 (18.75%) were strong, 6(18.75%) were moderate, and 20 (62.50%) were weak biofilm formers. Of 20 1/2b serotype strains, 3(15.00%) were strong, 8(40.00%) were moderate, and 9(45.00%) were weak biofilm formers. In case of 4b serotype, none of the strain could exhibit strong biofilm formation, whereas, 14(30.43%) strains showed moderate and 32(69.57%) showed weak biofilm formation.

No significant correlation was found between crystal violet OD (biofilm formation) and final cell density (coefficient of determination, r^2^ = 0.044). Serotype 1/2a strains particularly showed r^2^ value as 0.0012 (Fig 1a) between growth turbidity and crystal violet OD, whereas serotype 1/2b and 4b strains showed r^2^ value of 0.02 (Fig 1b) and 0.03 (Fig 1c), respectively.

In addition, biofilm-forming capabilities varied as per the source of isolation of the strain. Of 32 strains from clinical cases in animals, 11(34.38%) had moderate and 21(65.63%) had weak biofilm-forming ability. Of 18 strains tested from human clinical cases, 10(55.56%) were weak, and 8 (44.44%) were moderate biofilm formers. In case of strains from meat samples 2(14.29%) were moderate and 12(85.71%) were weak biofilm formers. None of the strain from animal clinical cases, human clinical cases, and meat sources could show strong biofilm formation. Of the strains isolated from milk and milk products, 9 (26.46%) were strong, 6(17.65%) moderate, and 19(55.88%) were weak biofilm formers. The strong biofilm-forming strains were isolated only from milk and milk products (Table 1).

**Table 1:** Strong, moderate, and weak biofilm-forming capabilities of *L*. *monocytogenes* isolates from different sources analyzed by microtiter plate assay.

Biofilm-forming capability				
Source of Isolates	Total	Strong	Moderate	Weak
**Animal clinical**	32	0(0.00%)	11(34.38%)	21(65.63%)
**Human clinical**	18	0(0.00%)	8(44.44%)	10(55.56%)
**Meat**	14	0(0.00%)	2(14.29%)	12(85.71%)
**Milk**	34	9(26.47%)	6(17.65%)	19(55.88%)
**Total**	**98**	**9 (9.18%)**	**27(27.55%)**	**62(63.26%)**

### SEM analysis of biofilm

**Fig 2:**
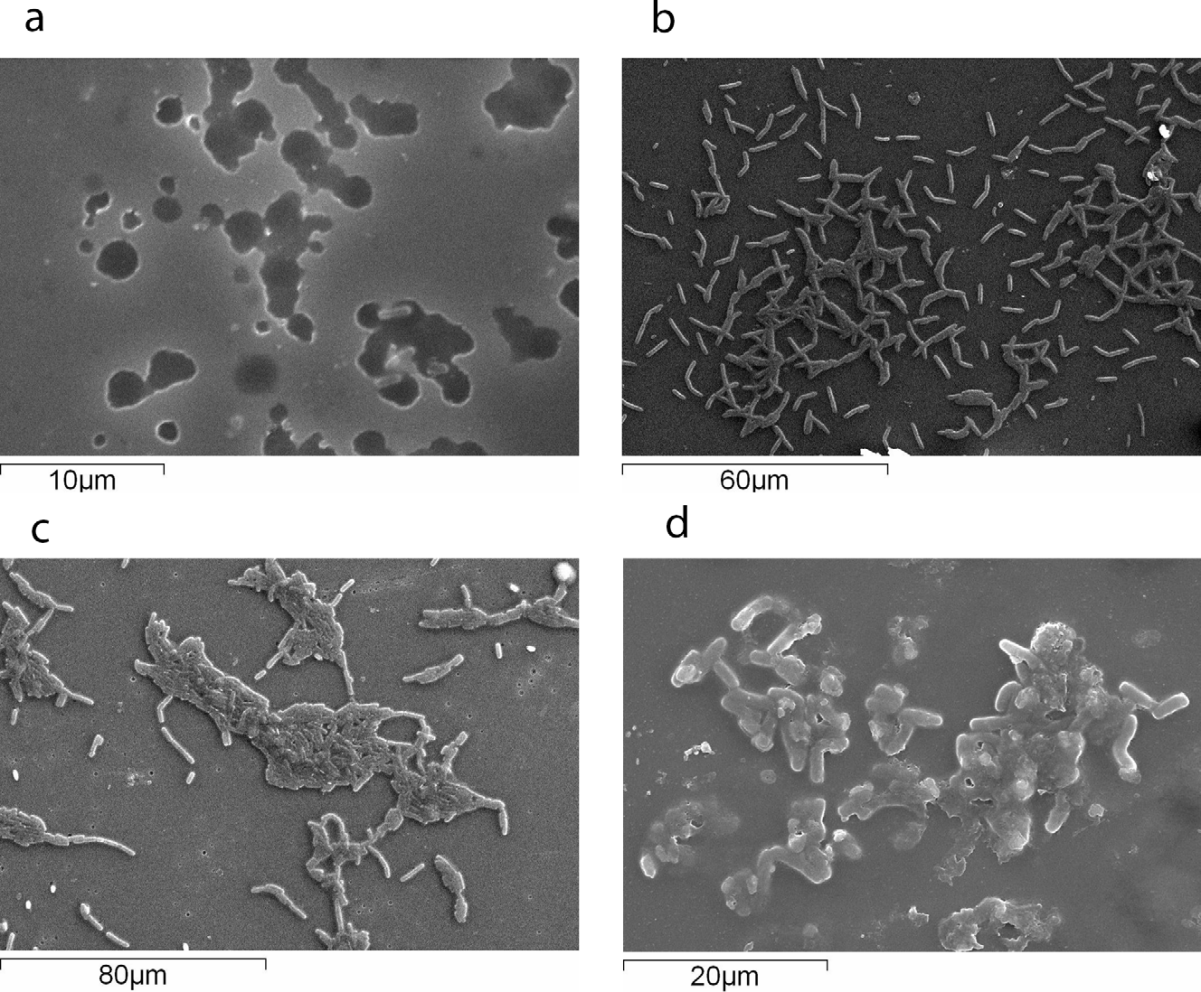
Scanning Electron Microscopy (SEM) of *L. monocytogenes* biofilm formation. *Listeria monocytogenes* strain was grown at 28°C in BHI on glass slides and observed after. (a) 2 h, adherence of *Listeria monocytogenes* to glass surface; (b) 6 h, adherent cells multiplied and began multilayer formation within 6 h of deposition; (c) 12 h,a mature biofilm was observed; (d) 24 h, cells surrounded by matrix could be seen.

Biofilm formation by listerial isolates was studied by SEM. The strongest biofilm-forming strain (ILCC306) was observed for its adherence, multiplication, and biofilm-forming capability. This strain could adhere to glass surface within 2 h, showing an initial attachment step (Fig 2a), followed by a firm adherence and the subsequent multiplication of cells increasing the biofilm (Fig 2b, 2c and 2d). The adherent cells multiplied and began multilayer formation within 6 h of deposition. After 12 h, a mature biofilm was observed, and after 24 h, cells surrounded by matrix could be seen. Listerial cells found to be embedded in the biofilm matrix at 24 h.

**Fig 3:**
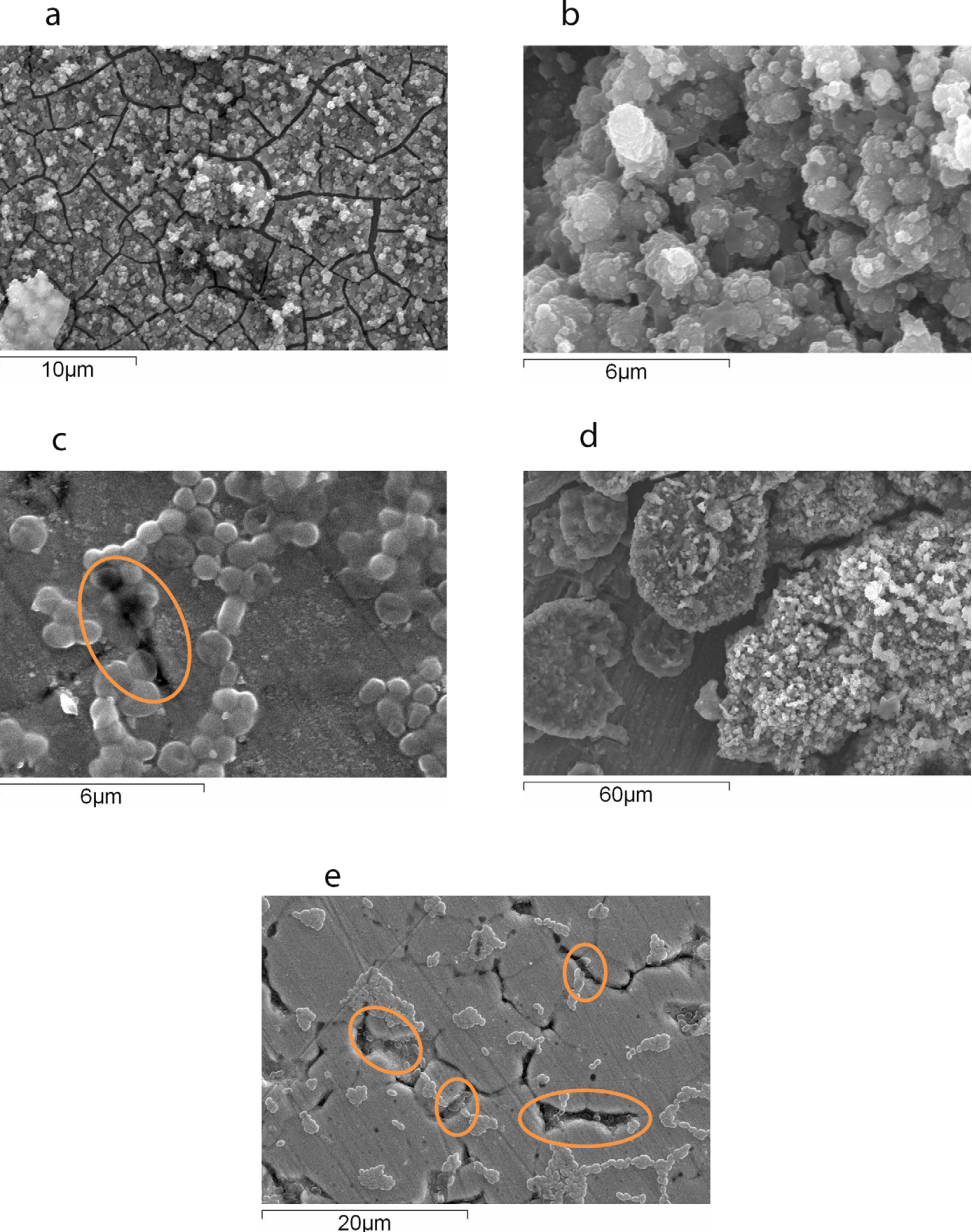
SEM of *Listeria monocytogenes* ILCC306 on different industrially important surfaces. **(a)** *L*. *monocytogenes* ILCC306 on PVC pipe after 24 h. Multilayered and mat-like biofilms were observed; **(b)** *L*. *monocytogenes* ILCC306 on ceramic tiles after 24 h. The cells were found to be aggregated all over the surfaces of ceramic tiles; **(c)** *L*. *monocytogenes* ILCC306 on stainless steel (SS304) after 24 h. Biofilm aggregated near suture; **(d)** *L*. *monocytogenes* ILCC306 on stainless steel suture (artificially made) after 24 h; **(e)** *L*. *monocytogenes* ILCC306 on HDPE plastic after 24 h. The circled area shows biofilm rooted in the sutures; bacterial growth can be seeninside the sutures and aggregates formation toward the surfaces.

SEM was also performed to observe the association of ILCC306 with different industrially important surfaces. Isolates were allowed to form a biofilm on stainless steel (SS304), high-density polyethylene (HDPE) plastic, PVC pipes, and ceramic tiles. Biofilm formation on all the surfaces was observed following 24 h incubation period. Industrial surfaces were found to contain a number of sutures and aggregates of cells gathered at sutures. Multilayered and mat-like biofilms were observed on the PVC pipes (Fig 3a). The cells were found to be aggregated all over the surfaces of ceramic tiles (Fig 3b). A higher biofilm formation was observed near the sutures than on the plain surface area of SS304 (Fig 3c). Comparatively, SS304 with artificial sutures showed higher biofilm formation with three-dimensional structures (Fig 3d). In case of HDPE material, biofilm rooted in the sutures could be seen (Fig 3e). Cells adhered inside the suture forming aggregates towards the surface. Interestingly, the morphology of the listerial cell, shorter rod was observed on the stainless steel surface.

### Quantitative biofilm formation assay

The biofilm-forming ability of the isolates was further analyzed by determining the actual number of cells present in the biofilm. Six representative strains from each type (strong, medium, and weak biofilm formers) were considered for the study to differentiate the biofilm-forming capability. Enumeration studies supported the data obtained from the microtiter plate assay, showing 570±127 × 10^3^ cells/cm^2^ by strong biofilm formers, followed by 33±26 × 10^3^ cells/cm^2^ by moderate biofilm former, and 5±3 × 10^3^ cells/cm^2^ by weak biofilm former.

### Determination of hydrophobicity

The HI of strong, moderate, and weak biofilm formers was determined by using n-hexadecane. Accordingly, although low, significant (P<0.05) difference was observed as per the biofilm- forming capability. The HI of strong biofilm formers was observed to be 29.06 ±1.31%, that of moderate biofilm former was 27.13 ±0.91%, whereas, that of weak biofilm former was 23.67±1.22%.

### Fatty acid profiles

Total fatty acid profiles of strong, moderate, and weak biofilm-forming strains were determined by the MIDI automated gas chromatographic instrument to observe whether there was any variation. Comparing the sample strains with reference standards, cell surface fatty acids ranging from 12:0 dodecanoic fatty acids to 20:0 eicosanoic fatty acids were identified and their relative amounts were estimated. The percentage of iso-tetradecanoic, iso- and anteiso-pentadeconic acid, iso- and anteiso-hexadecanoic acid, iso- and anteiso-ocatdecanoic acid was highest in strong biofilm former, followed by moderate biofilm former, and then weak biofilm former strains. Both anteiso- and iso-dodeacanoic acids were absent in the strong biofilm former. It was interesting to note that the amount of iso-C_14:0_, anteiso-C_15:0_, and iso-C_16:0_ fatty acids increases with increase in biofilm-forming capability of the *L*. *monocytogenes* isolates. However, some were significantly predominant among them, whereas, some occurred in a lesser amount. The predominant fatty acids present in all *L*. *monocytogenes* strains were anteiso-15:0 and anteiso-17:0 fatty acids (Table 2).

**Table 2:** Fatty acid profile of the strong, moderate, and weak biofilm-forming isolates as analyzed by the FAME analysis.

	Weak Average % ± SD	Moderate Average % ± SD	Strong Average % ± SD
Iso-C_12:0_	0.22 ± 0.01	0.51± 0.09	0
Anteiso-C_12:0_	0.53 ± 0.48	0.57 ± 0.12	0
Iso-C_13:0_	0	0	0
Anteiso-C_13:0_	0.22 ± 0.02	0.66± 0.16	0.15 ± 0.03
Iso-C_14:0_	0.33 ± 0.05	0.46 ± 0.06	0.52 ± 0.03
Anteiso-C_14:0_	0.42 ± 0.21	1.04 ± 0.26	0.29 ± 0.06
Iso-C_15:0_	5.76 ± 1.74	7.93 ± 0.53	7.79 ± 0.87
Anteso-C_15:0_	34.29 ± 2.63	37.18 ± 1.46	41.58 ± 1.55
Iso-C_16:0_	1.82 ± 0.68	2.51 ± 0.12	2.93 ± 0.36
Anteiso-C_16:0_	3.14 ± 1.78	3.43 ± 1.05	2.43 ± 0.39
Iso-C_17:0_	3.79 ± 2.08	4.78 ± 0.20	4.37 ± 0.81
Anteiso-C_17:0_	36.5 ± 2.74	42.4 ± 1.84	38.63 ± 1.41
18:1 w9c	1.66 ± 2.53	1.49 ± 0.66	1.11 ± 1.39
Iso-C_18:0_	0.73 ± 0.74	0.59 ± 0.16	0.76 ± 1.04
Anteiso-C_19:0_	0.33 ± 0.16	0.54 ± 0.01	0.23 ± 0.03
20:2 w6,9c	1.12 ± 1.86	0	1.41 ± 0.23

## Discussion

*Listeria monocytogenes* poses a serious threat to public health, and the majority of cases of human listeriosis are associated with the consumption of contaminated food. The presence of biofilms in production lines is related to the contamination of RTE products with *L*. *monocytogenes*, as biofilms protect cells from chemical sanitizers [36]. The biofilm-forming capability of *L*. *monocytogenes* allows its persistence in the food processing environment. Subsequently, such persisting cells may unknowingly get added in the food. Therefore, understanding the biofilm-forming capabilities of *L*. *monocytogenes* is of great interest. The present study was conducted to determine the biofilm-forming capability of *L*. *monocytogenes* strains isolated from different sources, belonging to different serotypes.

In this study, isolates of different serotypes from human clinical cases, animal clinical cases, milk, and meat were studied for their ability to form a biofilm. Although several researchers have used diluted nutrient medium or minimal medium to mimic environmental low-nutrient conditions, the laboratory based imitations in comparison to the actual natural environments is arguable. BHI broth has been extensively used to study the biofilm formation capabilities of *L*. *monocytogenes* [37–40], and reported best biofilm formation of *L*. *monocytogenes* in BHI broth than other media studied [41, 42]. Therefore, to determine the biofilm formation capabilities of *L*. *monocytogenes* at its best, BHI broth was used in this study. Additionally, there are few studies available that compare the biofilm formation of *L*. *monocytogenes* in different nutrient media. A study by Li et al. [42] observed higher biofilm formation capability in BHI broth followed by and Hsiang-Ning Tsai medium and LB broth. Stepanovic et al. [43] also reported better biofilm formation in BHI broth followed by Trypticase soya broth, then meat broth. These studies suggest, compared to BHI broth, lower biofilm formation in other nutrient media used for a particular strain. Only 9 out of 98 strains could exhibit strong biofilm formation as revealed by microtiter plate assay, whereas, a majority of the isolates showed weak or moderate biofilm-forming ability. The results of previous studies indicated that listerial cells were generally weak to moderate biofilm producers [20, 21, 35]. In this study, it was interesting to note that all the stronger biofilm-forming strains were isolated from milk and milk products. Harsh processing conditions applied during the processing of milk and milk products might be responsible for the selection of strong biofilm-producing strains. None of the strain from animal clinical cases, human clinical cases, and meat exhibited strong biofilm formation.

As serotypes have been revealed as one of the important strain-differentiating factor, particularly helpful in epidemiology, several researchers have correlated serotypes with different properties of *L*. *monocytogenes*. In case of listerial biofilms, the previously available data relating phylogenetic division, serotype, and biofilm formation remained inconclusive [18, 20, 21]. Some researchers found the correlation among particular serotype of *L*. *monocytogenes* and their biofilm forming capabilities, some could not find any correlation [20, 21]. In this study, we could not observe any relation with serotypes and their biofilm-forming ability. This suggested that there might not be any association between serotype and biofilm-forming capability, whereas, the observed associations could be because of random strong biofilm-forming strains in independent studies. Apparently, the biofilm formation of *L*. *monocytogenes* was strain dependent. In addition, we tried to correlate between biofilm-forming ability and final cell density. However, in line with Djordjevic [20], biofilm-forming ability was found independent of final cell density.

Previous studies showed that *L*. *monocytogenes* did not produce a sufficient amount of polymeric substances unlike *Pseudomonas* spp., *Staphylococcus*, or other biofilm formers. Therefore, to determine the composition of *L*. *monocytogenes* biofilm, bacterial cells in the biofilm were enumerated. It was found that the stronger biofilms had a higher number of cells than those in the weaker biofilm.

SEM studies were performed to observe progressive biofilm formation and its structure. *L*. *monocytogenes* strains could adhere after 0–2 h, the adhered cells could grow after 3–6 h, form two-dimensional mat after 7–12 h, and form a mature biofilm after 12–24 h. Previously, several different biofilm morphologies such as dense three-dimensional structure [21], honeycomb-like structure [44], mushroom-like or knitted chain structure [20, 45] non-organized and aggregated structure [23] have been observed in case of *L*. *monocytogenes*. However, in this study no defined structure was observed and the cells were found to adhere as a mat. Moreover, *L*. *monocytogenes* formed a biofilm on all the industrially important surfaces such as SS304, PVC, ceramic tiles, HDPE plastics, and glass. However, unlike many other previously reported studies which reported three-dimensional biofilm, except for SS304 with artificial suture, we observed only two-dimensional biofilm [46]. The probable reason could be the continuous shaking conditions used during the study. Most of the previously reported studies for listerial biofilm were conducted at static conditions, which is not always the case in the food processing environment. The shaking may have continuously detached the cells that would have been added on the outer layers of the film.

Electron microscopy studies also revealed that the biofilm was always situated near microscopic sutures and ridges. The biofilm rooted deeply in rifts could adhere the cells more strongly, resisting the swirl formed by the broth during growth under shaking condition. Therefore, these protected cells might have resisted the detachment of cells or sutures that could have acted as physical barriers to the flow of the liquid medium. To confirm this, biofilm formation was studied on an artificially induced suture on SS304. When observed under an electron microscope, three-dimensional biofilm could be observed. Such sutures or cracks could be the intrinsic properties of stainless steel and could be generated because of their repeated exposure to high temperature during clean-in-place procedures. Biofilm in such a minute sutures can act as a strong base, and it would be very difficult to remove even by a scrubber. Overall, biofilm formation on such industrially important surfaces is a matter of concern.

During electron microscopy studies, a slight change was noted in the morphology of *L*. *monocytogenes* cells that adhered to industrially important surfaces. Cells were comparatively shorter as compared to its normal, short-rod morphology. Such an altered morphology of *L*. *monocytogenes* has previously been reported because of the suboptimum conditions of the environment or different stresses [47, 48]. In contrast, Wen et al. [49] reported that such a change could be because of the transition from the log to the long-term survival phase.

It is known that the process of biofilm formation is greatly influenced by several factors such as the type of strain and growth conditions. Many researchers have tried to relate total fatty acids composition about the bacterial biofilm-forming capabilities [26–28]. We attempted to determine whether any differences exist in total fatty acid profiles of strong, moderate, and weak biofilm-forming isolates. Notably, the amount of iso-C_14:0_, anteiso-C_15:0_, and iso-C_16:0_ fatty acids increased with the increase in biofilm-forming capability. Fatty acids iso-C_15:0_, anteiso-C_15:0_, and anteiso-C_17:0_ formed the characteristic profile of *L*. *monocytogenes* [50]. Recently, such fatty acids have been suggested to play a role in the adhesion characteristics of *L*. *monocytogenes* [51]. Gianotti et al. [52], have reported that adherent cells contain a higher amount of C_16:0_ and C_18:0_ than planktonic *L*. *monocytogenes* cells. Moreover, the quantity and composition of fatty acids such as anteiso-C_17:0_ and anteiso-C_15:0_ have been reported to alter easily because of the adaptation of *L*. *monocytogenes* in different conditions [29, 53]. Such increase in the fatty acid content in relation to an increased hydrophobicity or biofilm formation has been reported for *P*. *aeruginosa* [26] and *S*. *aureus* [54]. The presence of the increasing amount of total fatty acids (iso-C_14: 0_, anteiso-C_15:0_, and iso-C_16:0_) as per the intensity of the biofilm suggested that fatty acids played a significant role in influencing the biofilm-forming capabilities of *L*. *monocytogenes*. To determine the specificity and exact function of such fatty acid toward biofilm formation, detailed studies are necessary.

In conclusion, this study revealed that *L*. *monocytogenes* could form biofilms on industrially important surfaces within 24 h. The correlation between serotype and biofilm formation remained inconclusive; it is apparently depended on individual strains. The biofilm formation by *L*. *monocytogenes* was independent of the growth. The sutures present on the industrial food-contact surfaces provided a site to root the biofilm. The number of *L*. *monocytogenes* cells at a given time and place defined the intensity of the biofilm. Certain fatty acids may play a significant role in determining the fate of biofilm. Therefore, biofilm-forming strains were more likely to persist in food processing environment that might result in the contamination of food. Although most of the *L*. *monocytogenes* cells formed moderate to weak biofilms, food industrial environment might harbor multicellular biofilm, increasing the prevalence of *L*. *monocytogenes*. Biofilm formation by such pathogens in the food chain is a matter of concern.

## Supporting Information

S1 Table List of *Listeria monocytogenes* isolates used in this study. All the isolates were obtained from Indian *Listeria* Culture Collection (ILCC), ICAR Research Complex for Goa, Goa, India. The cultures are now available with Indian *Listeria* Culture Collection, Centre of Excellence and Innovation in Biotechnology on “Translation Centre for Molecular Epidemiology of *Listeria monocytogenes*”.
(PDF)
