# Supplementary material for: Biofilm-Forming Abilities of Listeria monocytogenes Serotypes Isolated from Different Sources
Source: PLoS One. 2015 Sep 11;10(9):e0137046. doi: 10.1371/journal.pone.0137046 (PMC4567129; doi:10.1371/journal.pone.0137046)
Supplement: S1 Table — All the isolates were obtained from Indian Listeria Culture Collection (ILCC), ICAR Research Complex for Goa, Goa, India. The cultures are now available with Indian Listeria Culture Collection, Centre of Excellence and Innovation in Biotechnology on “Translation Centre for Molecular Epidemiology of Listeria monocytogenes”. (PDF) [file pone.0137046.s001.pdf]

S1 Table. List of isolates used in this study. All the isolates were obtained from Indian Listeria Culture Collection (ILCC), ICAR Research Complex for Goa, Goa, India.

The cultures are now available with Indian *Listeria* Culture Collection, Centre of Excellence and Innovation in Biotechnology on "Translation Centre for Molecular Epidemiology of *Listeria monocytogenes"*.

ILCC Id	Serotype	Source	Place of Isolation	Year of isolation	Reference
ILCC005	1/2a	Animal	Goa	2001	Unpublished
ILCC026	4b	Human	Goa	2006	Kalekar et al., 2011
ILCC027	1/2b	Human	Goa	2006	Kalekar et al., 2011
ILCC040	1/2b	Human	Kolhapur	2001	Unpublished
ILCC041	1/2a	Animal	Mumbai	2005	Unpublished
ILCC041a	1/2a	Animal	Mumbai	2005	Unpublished
ILCC046	4b	Animal	Mumbai	2005	Unpublished
ILCC047	4b	Animal	Mumbai	2010	Raorane et al., 2014
ILCC048	4b	Animal	Mumbai	2010	Raorane et al., 2014
ILCC049	4b	Animal	Mumbai	2010	Raorane et al., 2014
ILCC050	4b	Animal	Mumbai	2010	Raorane et al., 2014
ILCC094	4b	Human	Pondicherry	2006	Kalekar et al., 2011
ILCC094a	4b	Human	Pondicherry	2006	Kalekar et al., 2011
ILCC095	1/2b	Human	Agra	2012	Unpublished
ILCC097	1/2b	Human	Agra	2012	Unpublished
ILCC098	4b	Human	Agra	2006	Kalekar et al., 2011
ILCC099	1/2b	Human	Pondicherry	2006	Unpublished

ILCC115	4b	Animal	Izatnagar	2006	Shakuntala et al., 2006
ILCC140	1/2b	Human	Kolhapur	2006	Kalekar et al., 2011
ILCC142	4b	Human	Mumbai	2005	Kalekar et al., 2011
ILCC143	4b	Animal	Izatnagar	2006	Shakuntala et al., 2006
ILCC144	4b	Animal	Goa	2001	Unpublished
ILCC146	4b	Animal	Mumbai	2005	Unpublished
ILCC147	4b	Animal	Mumbai	2005	Unpublished
ILCC149	1/2a	Animal	Mumbai	2005	Unpublished
ILCC151	1/2a	Meat	Mumbai	2004	Unpublished
ILCC154	4b	Meat	Goa	2006	Doijad et al., 2010
ILCC155	1/2a	Meat	Goa	2006	Doijad et al., 2010
ILCC158	1/2a	Meat	Goa	2006	Doijad et al., 2010
ILCC159	1/2a	Meat	Goa	2006	Doijad et al., 2010
ILCC160	1/2a	Meat	Goa	2006	Doijad et al., 2010
ILCC161	4b	Meat	Goa	2006	Doijad et al., 2010
ILCC163	1/2a	Meat	Goa	2006	Doijad et al., 2010
ILCC164	1/2a	Meat	Goa	2006	Doijad et al., 2010
ILCC165	4b	Animal	Kolhapur	2001	Unpublished
ILCC166	1/2a	Meat	Goa	2006	Doijad et al., 2010
ILCC171	4b	Animal	Nagpur	2006	Kalorey et al., 2008
ILCC172	4b	Animal	Nagpur	2006	Kalorey et al., 2006
ILCC173	4b	Animal	Nagpur	2006	Kalorey et al., 2008
ILCC174	4b	Animal	Nagpur	2006	Kalorey et al., 2006
ILCC175	4b	Animal	Anand	2009	Yadava and Roy 2009

ILCC177	4b	Animal	Anand	2009	Yadava and Roy 2009
ILCC180	4b	Human	Goa	2009	Kalekar et al., 2011
ILCC181	4b	Animal	Anand	2009	Yadava and Roy 2009
ILCC182	4b	Animal	Anand	2009	Yadava and Roy 2009
ILCC243	4b	Animal	Anand	2009	Yadava and Roy 2009
ILCC249	4b	Milk	Nagpur	2008	Kalorey et al., 2008
ILCC264	4b	Milk	Nagpur	2008	Kalorey et al., 2008
ILCC276	4b	Animal	Izatnagar	2001	Shakuntala et al., 2006
ILCC283	1/2b	Milk	Mumbai	2004	Unpublished
ILCC284	1/2b	Milk	Mumbai	2004	Unpublished
ILCC285	1/2b	Milk	Mumbai	2004	Unpublished
ILCC289	1/2b	Milk	Mumbai	2004	Unpublished
ILCC291	1/2b	Milk	Mumbai	2004	Unpublished
ILCC297	1/2b	Milk	Mumbai	2004	Unpublished
ILCC298	1/2b	Milk	Mumbai	2004	Unpublished
ILCC299	1/2a	Milk	Mumbai	2004	Unpublished
ILCC300	1/2b	Milk	Mumbai	2004	Unpublished
ILCC301	1/2a	Milk	Mumbai	2004	Unpublished
ILCC302	1/2a	Milk	Mumbai	2004	Unpublished
ILCC303	1/2a	Milk	Mumbai	2004	Unpublished
ILCC304	1/2a	Milk	Mumbai	2004	Unpublished
ILCC306	1/2a	Milk	Mumbai	2004	Unpublished
ILCC307	1/2a	Milk	Mumbai	2004	Unpublished
ILCC309	1/2b	Milk	Mumbai	2004	Unpublished

ILCC312	1/2a	Milk	Mumbai	2004	Unpublished
ILCC317	1/2a	Milk	Goa	2007	Parihar et al., 2007
ILCC325	1/2a	Milk	Goa	2007	Parihar et al., 2007
ILCC336	1/2a	Milk	Goa	2007	Parihar et al., 2007
ILCC373	1/2a	Milk	Kolhapur	2010	Doijad et al., 2011
ILCC379	1/2a	Milk	Kolhapur	2010	Doijad et al., 2011
ILCC395	1/2b	Milk	Kolhapur	2001	Unpublished
ILCC400	1/2a	Milk	Goa	2009	D’costa et al., 2012
ILCC405	1/2a	Milk	Goa	2009	D’costa et al., 2012
ILCC416	1/2b	Milk	Goa	2009	Dcosta et al., 2012
ILCC419	1/2b	Milk	Goa	2009	D’costa et al., 2012
ILCC468	4b	Meat	Izatnagar	2001	Barbuddhe et al., 2000
ILCC470	4b	Meat	Izatnagar	1997	Barbuddhe et al., 2000
ILCC471	4b	Meat	Izatnagar	1997	Barbuddhe et al., 2000
ILCC474	4b	Meat	Izatnagar	1997	Barbuddhe et al., 2000
ILCC491	4b	Animal	Izatnagar	1997	Barbuddhe et al., 2000
ILCC492	4b	Animal	Mumbai	2005	Unpublished
ILCC493	4b	Animal	Izatnagar	1997	Barbuddhe et al., 2000
ILCC494	4b	Animal	Nagpur	2006	Kalorey et al., 2008
ILCC496	4b	Animal	Izatnagar	2006	Shakuntala et al., 2006
ILCC498	4b	Animal	Izatnagar	2006	Shakuntala et al., 2006
ILCC499	4b	Animal	Izatnagar	2006	Shakuntala et al., 2006
ILCC519	1/2a	Milk	Kolhapur	2010	Doijad et al., 2011
ILCC530	1/2a	Milk	Kolhapur	2010	Doijad et al., 2011

ILCC531	1/2a	Milk	Goa	2007	Parihar et al., 2007
ILCC535	1/2a	Milk	Goa	2009	D'costa et al., 2012
ILCC540	1/2a	Milk	Goa	2009	D'costa et al., 2012
ILCC557	4b	Human	Mumbai	2005	Kalekar et al., 2011
ILCC559	1/2b	Human	Nagpur	2008	Unpublished
ILCC562	4b	Human	Mumbai	2005	Kalekar et al., 2011
ILCC564	4b	Human	Mumbai	2005	Kalekar et al., 2011
ILCC567	4b	Human	Mumbai	2005	Kalekar et al., 2011
ILCC569	1/2b	Human	Nagpur	2008	Unpublished

References

Barbuddhe SB, Malik SVS, Bhilegaonkar KN, Kumar P, Gupta LK. Isolation of *Listeria monocytogenes* and anti-listeriolysin O detection in sheep and goats. Small Rum Res. 2000; 38:151-5. doi:10.1016/S0921-4488(00)00155-3

D'Costa D, Bhosle SN, Dhuri RB, Kalekar S, Rodrigues J, Doijad SP, Barbuddhe SB. The occurrence and characterization of *Listeria* species isolated from milk production chain. Milchwissenchaft. 2012; 67: 43-6.

Doijad, S. P., Vaidya, V., Garg, S., Kalekar, S., Rodrigues J., D'Costa, D., Bhosle, S. and Barbuddhe S.B. Isolation and characterization of *Listeria* species from raw and processed meats. J Vet Public Hlth. 2010; 8(2): 83-8.

Doijad, S. P., Barbuddhe S.B., Garg, S., Kalekar, S., Rodrigues J., D'Costa, D., Bhosle, S. and Chakraborty, T. Incidence and genetic variability of *Listeria* species from three milk processing plants. Food Cont. 2010; 22: 1900-04. doi:10.1016/j.foodcont.2011.05.001

Kalekar S, Rodrigues J, D'Costa D, Doijad SP, Jangam AK, Malik SVS, Kalorey DR, Rawool DB, Hain T, Chakraborty T, Barbuddhe SB. Genotypic characterization of *Listeria*

monocytogenes isolated from humans in India. Ann Trop Med Parasitol. 2011; 105(5):351-8. doi: 10.1179/1364859411Y.0000000023.

Kalorey DR, Kurkure NV, Warke SR, Rawool DB, Malik SVS, Barbuddhe SB. Isolation of pathogenic *Listeria monocytogenes* in faeces of wild animals in captivity. Comp Immunol Microbiol Infect Dis. 2006; 29(5-6):295-300. doi:10.1016/j.cimid.2006.07.002

Kalorey DR, Kurkure NV, Warke SR, Rawool DB, Barbuddhe SB. *Listeria* species in bovine raw milk: a large survey of Central India. Food Cont. 2008; 19:109-12. doi:10.1016/j.cimid.2006.07.002.

Parihar VS, Barbuddhe SB, Chakurkar EB, Danielson-Tham M-L, Tham W. Isolation of *Listeria* species from farm bulk milk at the receiving dairy plant and cervico-vaginal samples from dairy cows. Indian J Compa Microbiol Immunol Infect Dis. 2007; 28: 53-5.

Raorane A, Doijad S, Katkar S, Pathak A, Poharkar K, Dubal ZB, Barbuddhe SB. Prevalence of *Listeria* spp. in Animals and Associated Environment. Adv Anim Vet Sci. 2014; 2 (2): 81–5.

Shakuntala I, Malik, SVS, Barbuddhe SB, Rawool DB. 2006. Isolation of Isolation of *Listeria monocytogenes* from buffaloes with reproductive disorders and its confirmation by polymerase chain reaction. Vet Microbiol. 2006; 117(2-4): 229-34. doi:10.1016/j.vetmic.2006.06.018

Yadava MM, Roy A. Prevalence of *Listeria* spp including *Listeria monocytogenes* from apparently healthy sheep of Gujarat State, India. Zoonoses Public Health. 2009; 56(9-10):515-24. doi: 10.1111/j.1863-2378.2008.01201.x.
